# Placental Expression of Bile Acid Transporters in Intrahepatic Cholestasis of Pregnancy

**DOI:** 10.3390/ijms221910434

**Published:** 2021-09-28

**Authors:** Edgar Ontsouka, Alessandra Epstein, Sampada Kallol, Jonas Zaugg, Marc Baumann, Henning Schneider, Christiane Albrecht

**Affiliations:** 1 Institute of Biochemistry and Molecular Medicine, Faculty of Medicine, University of Bern, Bühlstrasse 28, 3012 Bern, Switzerland; edgar.ontsouka@ibmm.unibe.ch (E.O.); alexandra.epstein@students.unibe.ch (A.E.); sampuak@gmail.com (S.K.); Jonas.zaugg@ibmm.unibe.ch (J.Z.); 2 Department of Obstetrics and Gyneacology, University Hospital, Effingerstrasse 102, 3010 Bern, Switzerland; Marc.Baumann@insel.ch (M.B.); henning.schneider@hispeed.ch (H.S.)

**Keywords:** intrahepatic cholestasis of pregnancy, human placenta, bile acids, transporters, pregnancy complications

## Abstract

Intrahepatic cholestasis of pregnancy (ICP) is a pregnancy-related condition characterized by increased maternal circulating bile acids (BAs) having adverse fetal effects. We investigated whether the human placenta expresses specific regulation patterns to prevent fetal exposition to harmful amounts of BAs during ICP. Using real-time quantitative PCR, we screened placentae from healthy pregnancies (*n* = 12) and corresponding trophoblast cells (*n* = 3) for the expression of 21 solute carriers and ATP-binding cassette transporter proteins, all acknowledged as BA- and/or cholestasis-related genes. The placental gene expression pattern was compared between healthy women and ICP patients (*n* = 12 each). Placental *SLCO3A1* (OATP3A1) gene expression was significantly altered in ICP compared with controls. The other 20 genes, including *SLC10A2* (ASBT) and *EPHX1* (EPOX, mEH) reported for the first time in trophoblasts, were comparably abundant in healthy and ICP placentae. *ABCG5* was undetectable in all placentae. Placental *SLC10A2* (ASBT), *SLCO4A1* (OATP4A1), and *ABCC2* mRNA levels were positively correlated with BA concentrations in ICP. Placental *SLC10A2* (ASBT) mRNA was also correlated with maternal body mass index. We conclude that at the transcriptional level only a limited response of BA transport systems is found under ICP conditions. However, the extent of the transcriptional response may also depend on the severity of the ICP condition and the magnitude by which the maternal BA levels are increased.

## 1. Introduction

Intrahepatic cholestasis of pregnancy (ICP) is a pregnancy-specific liver disorder affecting women worldwide, characterized by the onset of pruritus and elevation of serum bile acid (BA) concentrations. Abnormal metabolic profile including elevated cholesterolemia and maternal comorbidity, such as gestational diabetes, have been also reported [1,2,3]. Depending on the severity of the ICP, the occurring fetal adverse outcomes may include spontaneous preterm labor, fetal distress, and stillbirth [4,5,6]. Numerous factors (e.g., genetic, hormonal, and environmental conditions) are thought to be implicated in the pathogenesis of ICP. Among genetic factors, the mutations in the genes coding familial intrahepatic cholestasis protein-1 (*FIC1,* also named *ATPase phospholipid transporting 8B1 (ATP8B1)*), bile salt excretory protein (*BSEP*, also known as *ATP-binding cassette (ABC) subfamily B member 11 (ABCB11)*), and multi-drug-resistance protein 3 (*MDR3*, also named *ABCB4*) and altered activities of multi-drug-resistance-related protein 2 (*MRP2*, also named *ABCC2*) have been reported. Nonetheless, the underlying mechanism of ICP is not precisely known so far.

There is accumulating evidence linking the toxicity of BAs to adverse fetal and maternal outcomes. Therefore, the BA equilibrium within the maternal–fetal pool, which necessarily depends on a balanced BA transport across the placental barrier, is critical. In healthy pregnancies, the concentration of BAs is higher in the fetal than in the maternal circulation [7]. Therefore, vectorial transfer of BAs across the placenta mainly occurs from fetus to mother [8]. The transport from fetus to trophoblast is primarily mediated by anion/BA exchangers, whereas the transport from trophoblast to mother especially occurs via ABC transporter proteins, comparable to BA uptake and efflux in hepatocytes. In ICP, the transplacental gradient for BAs is reversed. Consequently, the net transport of BAs is directed towards the fetus, rather than being transported to the maternal side [9]. Surprisingly, fetal BAs are raised to a lesser extent than maternal BAs, implying a protective mechanism that limits BA uptake into the fetal circulation and/or enhances ATP-dependent carriers that transport against concentration gradients towards the maternal circulation [10]. Since the placenta is a vital organ, which plays a key role in fetal protection, it could be assumed that it would prevent fetal exposure to greater amounts of endobiotic toxic compounds, such as BAs. Although the exploration of human placental BA transport systems is clinically relevant, experimental studies reporting the placental gene expression of BA transporters and carriers are scarce or even lacking. This is particularly noticeable for transporters such as the solute carrier (SLC) family 10 member 2 (*SLC10A2*, also known as *apical sodium-dependent bile acid transporter (ASBT)*), epoxide hydrolase 1 (*EPHX1*, known in hepatic tissue as epoxide hydrolase *(EPOX)*), solute carrier organic anion transport protein 3A1 *(SLCO3A)*, *ATP8B1*, *ABCB11*, and *ABCB4* in health and disease conditions. An overview of currently known SLC and ABC transporters associated with BA transport is presented in Table 1.

Based on these premises, we carried out the current investigations on placental tissues and trophoblast cells obtained from healthy and ICP pregnancies. We hypothesized that (i) the expression of BA- and cholestasis-related transporters (Table 1) is altered in placentae from ICP patients as a protective response to higher maternal serum BA concentrations, and (ii) correlations exist between placental mRNA levels of BA transport proteins and maternal clinical data. Our objectives were to (i) determine in human placental tissues and trophoblast cells obtained from healthy pregnancies mRNA levels of 21 candidate solute carriers and ABC transporters, whose cellular localization and functional role in BA transport are already well established in hepatocytes and enterocytes; (ii) assess the effect of ICP (i.e., BA “overload”) on the placental mRNA levels of BA transport proteins; (iii) examine the association between placentally expressed BA- and cholestasis-related transport proteins and selected maternal clinical parameters as well as baby sex; and (iv) summarize the currently available knowledge on the BA transport machinery in human placenta.

## 2. Results

### 2.1. Study Participants

The clinical characteristics of pregnant women enrolled in ICP and control groups are summarized in Table 2. ICP patients had a comparable body mass index (BMI) to healthy pregnant controls. Maternal circulating BA levels were monitored only in case of serious suspicions of ICP. Thus, corresponding data are available only for women diagnosed to be positive for ICP.

### 2.2. Expression of Selected Solute Carriers and ATP-Dependent Transporter Genes with Affinity for BA- and Cholestasis-Related Molecules in Control Placentae and Trophoblast Cells

The primers listed in Table 3 (see Section 5), used for the amplification of corresponding genes in placental tissues and trophoblast cells, were validated on positive control tissues and cells (liver and hepatocyte cell line). They amplified the expected products in positive controls (data not shown).

As illustrated in Table 4, in human placental tissues, except *ABCG5*, whose mRNA transcripts were not found, the remaining 20 BA- and cholestasis-related transport genes were detected by qPCR and categorized as either expressed (defined in our studies as Ct values < 35) or only marginally expressed (defined as Ct values > 35).

Among the expressed genes, some exhibited, however, an inconsistent expression pattern. This was the case for *SLC10A1*, *SLC51A*, *SLCO1B1*, and *SLCO1B3* since the corresponding mRNA transcripts were detected only in 3/12, 2/12, 2/12, and 9/12 control specimens, respectively. The same applies for *ABCC1* and *ABCC4* expressions, as they were only detected in 5/12 and 6/12 control tissues, respectively (Table 4). The mRNA expression of *ABCB11* was marginal (Table 4). We did not find any sex-specific expression profiles of the transport proteins tested.

The gene expression profile of the investigated BA- and cholestasis-related transport proteins did not fully correspond between primary trophoblast cells and control placental tissues (Table 4). This is illustrated, for instance, by the dissimilar gene expression of *SLCO1A2* in placental tissue compared with trophoblast cells. In our samples, *ABCG5* expression was detected neither in control placental tissues nor in primary trophoblast cells.

### 2.3. Comparison of Transporter Expression in Patients and Healthy Controls

**Figure 1:**
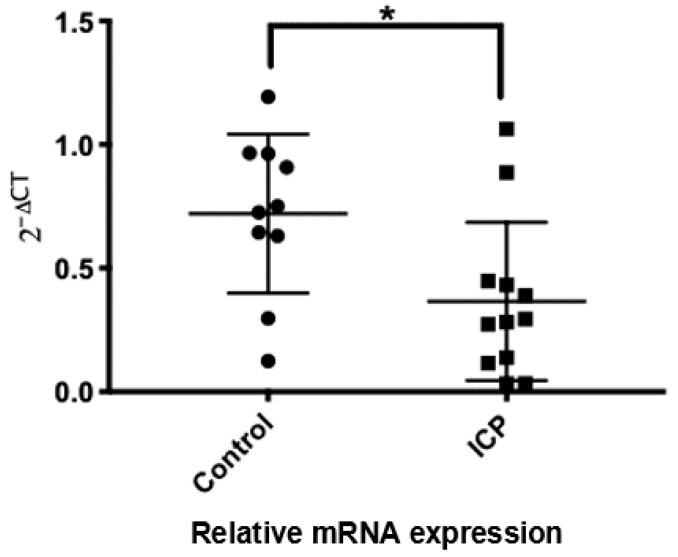
Comparative mRNA levels of SLCO3A1 in healthy and ICP placentae. Circle symbols represent healthy control placentae, and square symbols represent intrahepatic cholestasis of pregnancy (ICP). Real-time quantitative PCR and statistical evaluations of data are as described in the Section 5. * *p* = 0.0177.

In a next step, only the 13 BA- and cholestasis-related transport proteins, which were unequivocally detected in all heathy placentae, were compared with ICP placental tissues. Interestingly, we found that solely *SLCO3A1* mRNA expression was differentially expressed (*p* = 0.0177) in ICP placentae as compared with controls (Figure 1).

The remaining tested genes were unaltered (*p* > 0.05) by the ICP condition (Table 5). There were no sex-specific expression patterns found.

### 2.4. Correlation between Placental BA Transport Proteins and Clinical Parameters

We found significant positive relationships between placental *SLC10A2* mRNA levels and maternal BMI values (Table 6), independently of the maternal health status. In patients, *SLC10A2* mRNA levels were correlated with circulating BA levels (Table 6), whereas *SLCO4A1* and *ABCC2* mRNA levels were positively correlated with maternal serum BA concentrations (Table 6).

## 3. Discussion

### 3.1. Screening of Transporters in Placental Tissues and Trophoblast Cells

The present study describes the mRNA expression profile of important BA solute carriers and ABC transporters in placental tissues/cells and discusses their potential relevance as protective mechanisms preventing fetal exposure to excessive harmful BAs.

One of the main findings of the study is that, for the first time, the gene expression of *SLC10A2* is described in human placental tissue and trophoblast cells. SLC10A2/ASBT is a sodium-dependent transporter that exerts a crucial function in the enterohepatic circulation. It enables, at the apical membrane of enterocytes, the uptake of BAs from the intestinal lumen [11]. Previous findings have established that SLC10A2/ASBT abundance in the intestine is inversely correlated with maternal BA concentrations [12]. This is consistent with a role of this gene in preventing excess absorption of BAs into the portal circulation. By assuming that the (apical) localization of SLC10A2/ASBT is conserved in the trophoblast, the unexpected positive relationship of placental *SLC10A2* mRNA and maternal BA levels found in the present study appears intriguing. This finding does not argue for a role of placental SLC10A2/ASBT as a feto-protective mechanism against the deleterious effect of elevated maternal serum BA concentrations in ICP. Indeed, the herein observed positive correlation would suggest a parallel increase in placental *SLC10A2* expression with augmentation of maternal serum BA concentrations.

Nonetheless, the interpretation of the mentioned relationship requires some caution. Maternal blood samples (used for BA measurements during diagnosis) and placental tissue (for *SLC10A2* mRNA analysis after birth) have not been collected at identical time points. Hence, due to limitations based on our ethical approval, maternal BA levels at delivery (i.e., at the time of placenta tissue collection) could not be monitored. Thus, it is not certain whether the detected placental *SLC10A2* mRNA expression fully reflects its gene expression at the time of blood sampling.

Next, we identified mRNA transcripts of *SLC51B* in human placental tissue and in primary trophoblasts isolated from term control placentae. This finding is valuable since the available literature concerning trophoblast cells has reported so far only the gene expression of SLC51A/OST-α [13]. Considering the identification of *SLC51A* and *SLC51B* mRNA isoforms in the current study, it is likely that SLC51A/OST-α and SLC51B/OST-β are important in modulating BA fluxes across the placental barrier, similar to their role in other tissue/cell types [14,15].

An additional new finding in this study is based on the detection of mRNA expression of *EPHX1* (also called EPOX or mEH in hepatic tissue; see Table 1) in trophoblast cells. This result complements investigations by Coller et al., who, studying human placental tissue, described the presence of EPHX1/*EPOX* only in placental blood vessels and Hofbauer cells [16]. The discrepancy between these studies may be explained by the difference of the sensitivity of the methods employed. We applied the highly sensitive real-time quantitative PCR using cDNA from well-characterized isolated trophoblast cells, whereas Coller et al. used an immunostaining technique. Considering that EPHX1/EPOX operates as a sodium-dependent BA transporter in other mammalian cells [17,18], the identification of its mRNA in placental tissues and trophoblast cells may suggest a similar function in the human placenta. However, the lack of a significant correlation between the placental *EPHX1* gene expression and maternal BA concentrations could indicate a minor role of EPHX1/EPOX in controlling BA fluxes across the human placenta.

Considering that pregnant women with ICP are also prone to other metabolic features, especially dyslipidemia [2], *ABCG5* mRNA expression was determined. Surprisingly, we did not detect *ABCG5* mRNA expression, neither in our human placental tissues nor in the isolated trophoblast cells. These data are in contrast to findings in rats, where *Abcg5* and *Abcg8* mRNAs were detected [19]. Nonetheless, we did not find literature data reporting the expression of ABCG5/STSL in human placenta, implying that the placental expression of this membrane protein could be species specific.

### 3.2. Summary of the BA Transport Machinery in Human Placenta

**Figure 2:**
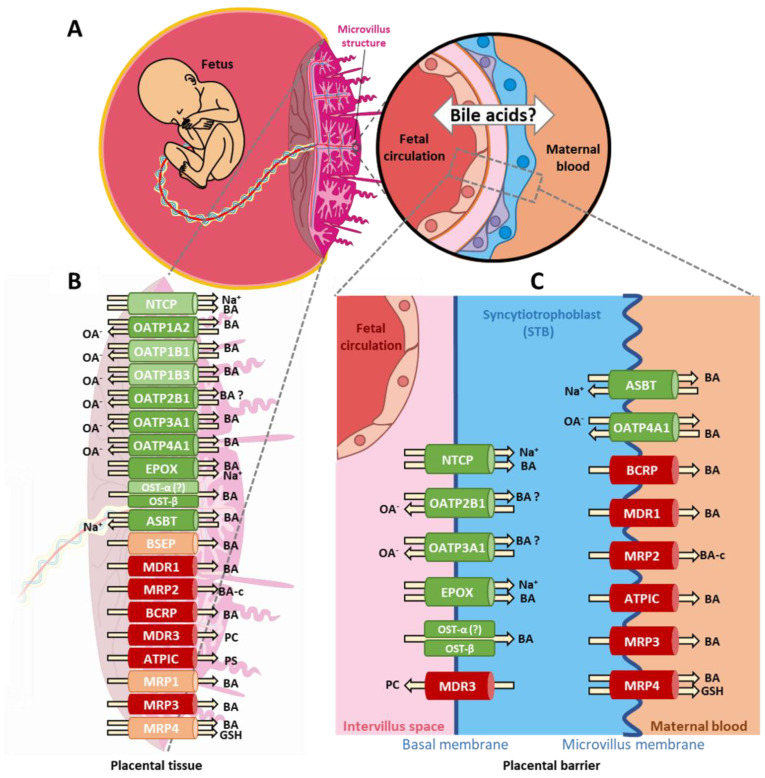
Schematic illustration of the bile acid transport machinery in human placenta. Fetal BAs are eliminated by transport across the placenta towards the maternal circulation (**A**). Arrows indicate the direction of substrate and cosubstrate transport exchange by the various transporter proteins across the human placenta (**B**) and across the trophoblast membranes, respectively (**C**), representing the critical part of the placental barrier. Details are described in the text. The gene and protein names of the depicted transporters are listed in Table 1 and Table 4. Abbreviations: BA: bile acids. BA-c: bile acid conjugates. OA^−^: organic anions. PS: phosphatidylserine. PC: phosphatidylcholine. BA (?) indicates uncertainties regarding the BA transport, while OST-α (?) indicates uncertainties regarding expression.

Given the expression profiles of BA transport proteins detected in healthy placental tissues and primary trophoblasts in the current study, we suggest the following scheme summarizing the BA transport machinery in human placental tissue (Figure 2). This overview is based on the assumption that the genes’ substrate affinity and polarization patterns are conserved and would therefore reflect findings in enterocytes/hepatocytes [14,15,20]. The fetal liver produces BAs as early as 12 weeks of gestation, which are eliminated as waste products by transporting them across the placenta towards the maternal circulation (Figure 2A). Conversely, maternal-originating BAs are also directed to the fetus through the placenta (Figure 2B,C). The transport proteins involved are located at the plasma membrane of the placental apical (microvillus) and basal layers (Figure 2C). The expressed (Ct value < 35) and consistently (present in all specimens) detected transport proteins in placental tissue and syncytiotrophoblasts are illustrated with symbols, filled in green and red colors, respectively. Faded colors indicate equivocally expressed transport proteins. The indicated localization of transport proteins in the trophoblast (Figure 2C), the substrate affinity, and the directionality of transport are according to existing literature under physiological conditions.

Notably, for a few transport proteins, such as SLCO1A2/OATP1A2 and SLCO1B3/OATP1B3, whose mRNA transcripts were absent in trophoblast cells used in this study, their placental expression is still a matter of controversy. Both absence [21] and presence [13,22,23] have been reported.

### 3.3. Comparison of ICP Versus Controls

Considering recently published data, stratifying the severity of ICP and its relationship to hazard risks of the prevalence of adverse perinatal outcomes [3], patients investigated in this study appeared to be “mildly” affected. The mean concentration of serum BAs was around 56 μmol/L, although a considerable interindividual variation was observed within the studied cohort. Of all 13 analyzed genes, solely the *SLCO3A1* mRNA expression was significantly altered in ICP compared with controls. It cannot be excluded that the clinical severity of ICP has an impact on the maternal and fetal regulation of the studied genes. Thus, depending on the severity of the disease and the cohort size, the expression of the genes might vary. The downregulation of *SLCO3A1* detected in this study per se seems interesting. SLCO3A1/OATP3A1 is an uptake transporter that has transport affinity for BAs, various steroid hormones, and others [21,24]. Among them are also prostaglandins, key regulators of myometrium contraction, which plays an important role, for example, during preterm labor associated with ICP [25].

Next, all subjects enrolled into the ICP cohort were treated with ursodeoxycholic acid (UDCA treatment). Contrary to the finding by Azzaroli et al. [26], who reported the gene-promoting effect of UDCA on *ABCC2* expression, we did not observe alterations in the *ABCC2* expression in this study. We were unable to determine whether UDCA treatment caused changes in the placental *SLCO3A1* mRNA expression or whether the altered expression pattern resulted from the effect of maternal BA. Moreover, further studies aiming to precisely identify the SLCO3A1/OATP3A1 localization (apical versus basal membranes) and functionality (e.g., with placental trophoblast cell-based Transwell^®^ system or ex vivo dual perfusion of the human placenta) are needed. They will help to draw firm conclusions regarding whether *SLCO3A1* gene expression downregulation constitutes a protective measure for the fetus exposed to maternal ICP.

## 4. Conclusions

Data reported in the current study indicate that the human placenta exhibits a limited response at the transcriptional level when the mother suffers from a “moderate” ICP condition. The newly identified gene expression of *SLC10A2* and *EPHX1* in human placenta tissue and trophoblasts was unaltered in ICP, while *SLC10A2* mRNA strongly correlated with both maternal BMI and BA levels. Nonetheless, given the relatively small cohort size of controls and ICP patients in the present study, the reported findings and interpretations may not be generalized unless confirmed in a larger cohort.

## 5. Materials and Methods

### 5.1. Human Placental Tissue and Trophoblast Cells

The study was approved by the ethics institutional review board of the Canton of Bern with an informed consent obtained from each participant prior to giving birth. The study was conducted in accordance with the Declaration of Helsinki. Pregnant women were under obstetrical care at the Department of Obstetrics and Gynecology, University Hospital, Bern, Switzerland. Placentae from healthy controls (*n* = 12) and ICP (*n* = 12) pregnancies were obtained after elective caesarean section or from spontaneous delivery between 2009 and 2011 after having obtained informed consent from the pregnant women. Placentae from healthy pregnancies were used. The clinical characteristics of pregnancies, whose placentae were investigated, are summarized in Table 2.

Upon serious suspicion, pregnant women were diagnosed for ICP following the routinely applied procedure at the University Hospital. The criteria of eligibility for the pregnant women’s inclusion to the ICP group include, among others, increased serum BA concentration in combination with pruritus. All ICP women were treated with appropriate doses of De-Ursil^®^ from diagnosis until delivery.

In addition to placental tissues, a cDNA pool of three independent trophoblast cell isolations was also tested. The procedure of trophoblast isolation has been previously described in detail [27].

### 5.2. RNA Extraction and Quantitative RT-PCR

Considering the heterogeneity of the human placenta, we standardized the placental tissue collection procedure for gene expression analysis [28]. Thus, each specimen analyzed was taken from the central area of the placenta. Total RNA was extracted from placental tissues using Trizol^®^ reagent (Thermo Fisher Scientific, Waltham, MA, USA). The concentrations of the extracted total RNA were calculated by measuring absorbance (A) at 260 nm. Purity was assessed by the A260/280 and A260/230 ratios, measured using a NanoDrop ^TM^ 1000 Spectrophotometer (Thermo Fisher Scientific, Waltham, MA, USA). Next, 2 μg of total RNA from each sample was reverse-transcribed using the GoScript ™ Reverse Transcriptase System (Promega, Madison, WI, USA) according to the manufacturer’s instructions. Real-time PCR was carried out on the ViiA 7 Real-Time PCR Detection System (Bio-Rad Laboratories Inc., Hercules, CA, USA) using an SYBR^®^ Green PCR master mix detection kit (Promega, Madison, WI, USA). The reaction conditions were as follows: an initial denaturation at 95 °C for 10 min, followed by 40 cycles of 95 °C for 15 s and 60 °C for 60 s, for melting curve temperature was increased from 60 °C to 95 °C with an increment rate of 0.5 °C every 0.05 s. The primers used for PCR amplification of the 21 currently known human BA- and cholestasis-related transport proteins are shown in Table 3. The relative mRNA expression of BA transport proteins was calculated by using the formula 2^−dCt^. For each individual sample, the dCt equates the difference between the Ct value of the transport protein of interest and the mean Ct value of the measured references. The latter were β-actin, YWHAZ, GAPDH, and ubiquitin.

### 5.3. Statistical Analysis

The statistical evaluation was performed using GraphPad Prism^®^ (GraphPad Software Inc., San Diego, CA, USA). All data are shown as mean ± SD. The gene expression data were analyzed for normality of distribution. Differences in the placental mRNA expression of targeted BA transport proteins between healthy controls and ICP patients were analyzed with unpaired *t*-test. The correlations of clinical data and gene expression of transport proteins were calculated with Pearson’s correlation test. The level of statistical significance was set at *p* ≤ 0.05.

## Figures and Tables

**Table 1:** Overview of currently known genes associated with bile acid transport.

Membrane Protein Class	Entry Number	Gene Name ^1^	Previous Symbols/Aliases ^1^	Approved Name ^1^
Solute carriers	Q14973 (NTCP_HUMAN)	*SLC10A1*	NTCP	Solute carrier family 10 member 1
	P46721 (SO1A2_HUMAN)	*SLCO1A2*	OATP, OATP1A2, OATP-A	Solute carrier organic anion transporter family member 1A2
	Q9Y6L6 (SO1B1_HUMAN)	*SLCO1B1*	SLC21A6/OATP1B1, OATP-C, LST-1	Solute carrier organic anion transporter family member 1B1
	Q9NPD5 (SO1B3_HUMAN)	*SLCO1B3*	SLC21A8/OATP1B3 OATP8,	Solute carrier organic anion transporter family member 1B3
	O94956 (SO2B1_HUMAN)	*SLCO2B1*	SLC21A9/ OATP2B1, OATP-B	Solute carrier organic anion transporter family member 2B1
	Q9UIG8 (SO3A1_HUMAN)	*SLCO3A1*	SLC21A11/ OATP3A1, OATP-D	Solute carrier organic anion transporter family member 3A1
	Q96BD0 (SO4A1_HUMAN)	*SLCO4A1*	SLC21A12/ OATP4A1, OATP-E	Solute carrier organic anion transporter family member 4A1
	P07099 (HYEP_HUMAN)	*EPHX1*	EPOX/EPHX1/ mEH	Epoxide hydrolase 1
	Q86UW1 (OSTA_HUMAN)	*SLC51A*	OST-α	Organic solute transporter subunit alpha
	Q86UW2 (OSTB_HUMAN)	*SLC51B*	OST-β	Organic solute transporter subunit beta
	Q12908 (NTCP2_HUMAN)	*SLC10A2*	ISBT/ASBT	Solute carrier family 10 member 2
ABC transporters	O95342 (ABCBB_HUMAN)	*ABCB11*	BSEP, PFIC2/ ABC16	ATP-binding cassette subfamily B member 11
	P08183 (MDR1_HUMAN)	*ABCB1*	MDR1/P-gp; CD243	ATP-binding cassette subfamily B member 1
	Q92887 (MRP2_HUMAN)	*ABCC2*	MRP2/CMOAT1	ATP-binding cassette subfamily C member 2
	Q9UNQ0 (ABCG2_HUMAN)	*ABCG2*	BCRP, MXR, ABCP, CD338	ATP-binding cassette subfamily G member 2
	P21439 (MDR3_HUMAN)	*ABCB4*	MDR3, PGY3/ MDR2, PFIC-3	ATP-binding cassette subfamily B member 4
	O43520 (AT8B1_HUMAN)	*ATP8B1*	FIC1, PFIC1/ATPIC, PFIC	ATPase phospholipid transporting 8B1
	Q9H222 (ABCG5_HUMAN)	*ABCG5*	STSL	ATP-binding cassette subfamily G member 5
	P33527 (MRP1_HUMAN)	*ABCC1*	MRP1/GS-X	ATP-binding cassette subfamily C member 1
	O15438 (MRP3_HUMAN)	*ABCC3*	MRP3, MOAT-D, cMOAT2, MLP2	ATP-binding cassette subfamily C member 3
	O15439 (MRP4_HUMAN)	*ABCC4*	MRP4/CFTR MOAT-B	ATP-binding cassette subfamily C member 4

^1^ Source: Gene nomenclature committee (https://www.genenames.org/data/genegroup/#!/group/752, accessed on 22 July 2021).

**Table 2:** Maternal clinical parameters.

Parameters	Controls (*n* = 12)	ICP (*n* = 12)
Maternal age, years	33.1 ± 4.1	30.5 ± 6.7
Gravidity	2.5 ± 1.4	2.5 ± 1.2
Parity	1.9 ± 0.9	1.5 ± 1.2
Gestational age, weeks	39.2 ± 0.8	37.9 ± 1.9
BMI, kg/m^2^	22.1 ± 2.4	23.1 ± 6.5
Baby gender (male/female)	6/6	6/6
Bile acid levels, μmol/L	n.a.	55.5 ± 61.7
De-Ursil^®^ treatment applied	*n* = 0	*n* = 12

Data are expressed as mean ± SD. ICP, intrahepatic cholestasis of pregnancy; BMI, body mass index; n.a., not analyzed.

**Table 3:** Primers used for gene amplification.

Gene	Forward Primer (5′-3′)	Reverse Primer (5′-3′)	Accession Number
*SLC10A1*/NTCP	GGAGGGAACCTGTCCAATGTC	CATGCCAAGGGCACAGAAG	NM_003049.3
*SLCO1A2*/OATP1A2	CACCACCTTCAGATACAT	GTAGATGACACTTCCTCAA	NM_005630.2
*SLCO1B1*/OATP1B1	CTTGTATTTAGGTAGTTTGA	CTTAGGAGTTATTCTGATAG	NM_019844.3
*SLCO1B3*/OATP1B3	ATAGAGCATCACCTGAGA	TCCACGAAGCATATTACC	NM_006446.4
*SLCO2B1*/OATP2B1	CACGAAGAAGCAGGATGG	CTGGGGAAGACTTTAATGAACT	NM_007256.4
*SLCO3A1*/OATP3A1	TTGTTGGGCTTCATCCCTCC	CGAAGGATTTGAGCGCGATG	NM_013272.3
*SLCO4A1*/OATP4A1	GAATACTAGGGGGCATCCCG	ATGGCAAAGAAGAGGACGCC	NM_016354.3
*EPHX1*/EPOX/mEH	CCCAAGGAGTAATCAGAGGGTG	ACATGGCTCCTGTACCTCAG	NM_000120.3
*SLC51A*/OST-α	CAGGTCTCAAGTGATGAA	CTTCGGTAGTACATTCGT	NM_152672.5
*SLC51B*/OST-β	GCTGCTGGAAGAGATGCTTTG	TTTCTTTTCTGCTTGCCTGGATG	NM_178859.3
*SLC10A2*/ASBT	CCTGGTACAGGTGCCGAAC	TGAGCGGGAAGGTGAATACG	NM_000452.2
*ABCB11*/BSEP	GACATGCTTGCGAGGACCTT	GGTTCGTGCACCAGGTAAGAA	NM_003742.2
*ABCB1*/MDR1	GCCAGAAACAACGCATTGCC	GGGCTTCTTGGACAACCTTTTC	NM_000927.4
*ABCC2*/MRP2	GATGCACAAAAGGCCTTCACC	GGAAACACTGGCCTGGAGCAT	NM_000392.4
*ABCG2*/BCRP	TGTGTTTATGATGGTCTGTTGGTC	GCTGCAAAGCCGTAAATCCA	NM_001257386.1
*ABCB4*/MDR3	GGACAGTGCTTCTCGATGGTC	TACAACCCGGCTGTTGTCTC	NM_000443.3
*ATP8B1*/FIC1	AGCAGTTTAAGAGAGCAGCC	TATGGCGAGCCACATCGTC	NM_005603.4
*ACGG5*/STSL	CCTCTCATCTTTGACCCCCG	CTCACGCGGTGGCTGAC	NM_022436.2
*ABCC1*/MRP1	TTAAGGTGTTATACAAGAC	GATGAGCAACTTTAAGAT	NM_004996.3
*ABCC3*/MRP3	GATACGCTCGCCACAGTCC	CAGTTGCCGTGATGTGGCTG	NM_003786.3
*ABCC4*/MRP4	CCATTGAAGATCTTCCTGG	GGTGTTCAATCTGTGTGC	NM_005845.4
*β-actin*	AACTCCATCATGAAGTGTGACG	GATCCACATCTGCTGGAAGG	NM_001101.5
*YWHAZ*	CCGTTACTTGGCTGAGGTTG	AGTTAAGGGCCAGACCCAGT	NM_145690.3
*GAPDH*	GCTCCTCCTGTTCGACAGTCA	ACCTTCCCCATGGTGTCTGA	NM_002046.7
*Ubiquitin*	TCGCAGCCGGGATTTG	GCATTGTCAAGTGACGATCACA	NM_021009

The primers used for amplification in placentae were designed with Beacon (Premier Biosoft, Palo Alto, CA, USA). They were validated for accurateness of amplification on positive tissues using immortalized liver carcinoma (HEPG2) cells. NTCP: sodium (Na)-taurocholate cotransporting polypeptide; OATP: organic anion transport; OST: organic solute transporter; ASBT: apical sodium-dependent bile acid transporter; EPHX1/EPOX/mEH: Epoxide hydrolase 1/microsomal epoxide hydrolase; BSEP: bile salt excretory protein; MDR: multi-drug-resistance protein; FIC1: familial intrahepatic cholestasis protein-1; MRP: multi-drug-resistance-related protein; ASBT: sodium-dependent bile acid transporter; ABC: ATP-binding cassette transporter; SLC: solute carrier protein; SLCO: solute carrier organic anion transporter; ATP8B1: ATPase phospholipid transporting 8B1.

**Table 4:** Expression of the investigated bile acid solute carriers and ABC transporters in control placentae and primary trophoblast cells.

Membrane Protein			mRNA Transcripts Detectable in	
Class	Protein Name	Gene Name	Placental Tissue (*n* = 12)	Trophoblasts (*n* = 3)
Solute carriers	NTCP	*SLC10A1*	3/12	all
	OATP1A2	*SLCO1A2*	all	n.d.
	OATP1B1	*SLCO1B1*	2/12	n.d.
	OATP1B3	*SLCO1B3*	2/12	n.d.
	OATP2B1	*SLCO2B1*	all	all
	OATP3A1	*SLCO3A1*	all	all
	OATP4A1	*SLCO4A1*	all	all
	EPOX/mEH	*EPHX1*	all	all
	OST-α	*SLC51A*	9/12	all
	OST-β	*SLC51B*	all	all
	ASBT	*SLC10A2*	all	all
ABC transporters	BSEP	*ABCB11*	Ct > 35	all
	MDR1	*ABCB1*	all	all
	MRP2	*ABCC2*	all	all
	BCRP	*ABCG2*	all	all
	MDR3	*ABCB4*	all	all
	FIC1	*ATP8B1*	all	all
	ABCG5	*ABCG5*	n.d.	n.d.
	MRP1	*ABCC1*	5/12	n.d.
	MRP3	*ABCC3*	all	all
	MRP4	*ABCC4*	6/12	all

NTPC: sodium (Na)-taurocholate cotransporting polypeptide; OATP: organic anion transport; OST: organic solute transporter; ASBT: apical sodium-dependent bile acid transporter; EPHX1: epoxide hydrolase 1; mEH/EPOX: microsomal epoxide hydrolase; BSEP: bile salt excretory protein; MDR: multi-drug-resistance protein; FIC1: familial intrahepatic cholestasis protein-1; MRP: multi-drug-resistance-related protein; ASBT: sodium-dependent bile acid transporter; ABC: ATP-binding cassette transporter; SLC: solute carrier protein; SLCO: solute carrier organic anion transporter; n.d.: not detected. The threshold of gene expression in this study was set at Ct < 35 amplification cycles. Details regarding procedures related to quantitative RT-PCR are given in Section 5.

**Table 5:** Summary of gene expression comparisons between ICP and controls.

Gene	*SLC10A2*	*ABCB1*	*ABCB4*	*ABCG2*	*ABCC2*	*ABCC3*	*ATP8B1*	*SLC51A*	*EPHX1*	*SLCO2B1*	*SLCO4A1*	*SLCO1A2*
*p*-value	0.99	0.86	0.73	0.84	0.37	0.47	0.78	0.33	0.37	0.71	0.43	0.44

Differences between ICP and controls were evaluated by using unpaired *t*-test. SLC: solute carrier protein; ABC: ATP-binding cassette protein; SLCO: solute carrier organic anion transporter; EPHX1: epoxide hydrolase 1; ATP8B1: ATPase phospholipid transporting 8B1.

**Table 6:** Summary of significant correlations.

	*SLC10A2*	*SLCO4A1*	*ABCC2*
BMI *	R2 = 0.27; *p* = 0.013		
Serum bile acids **	R2 = 0.58; *p* = 0.004	R2 = 0.34; *p* = 0.047	R2 = 0.70; *p* = 0.0007

Pearson’s correlation coefficients are shown. All pregnant women independent of their health status (*) or only patients (**) were included in the analysis. ABC: ATP-binding cassette protein; SLC: solute carrier protein; SLCO: solute carrier organic anion transporter.

## Data Availability

Not applicable.
